# Visual hallucinations in parkinson’s disease: Experience from a portuguese level 3 hospital

**DOI:** 10.1192/j.eurpsy.2021.1273

**Published:** 2021-08-13

**Authors:** J. Quarenta, T. Teixeira, S. Martins

**Affiliations:** Psychiatry Department, Centro Hospitalar entre o Tamega e Sousa EPE, Penafiel, Portugal

**Keywords:** Parkinson, visual hallucionations

## Abstract

**Introduction:**

Parkinson’s disease is a neurodegenerative pathology characterized by motor and non-motor symptoms. Hallucinations, especially visual ones, are frequent in this context, with an estimated prevalence of 16 to 40% and associated with a less favorable prognosis. These hallucinations can range from coarse formations to well-defined structures.

**Objectives:**

To assess the prevalence of visual hallucionations in a sample of patients diagnosed with Parkinson’s Disease.

**Methods:**

A cross-sectional descriptive study conducted in a Neurology Department of a Level III portuguese hospital. The sample included patients with Parkinson’s Disease observed in an outpatient Neurology appointment between October 1 and December 9, 2019.

**Results:**

In the period considered, 100 patients with Parkinson’s disease were observed, 65% male, with an average age of 69 years. In 11% of the patients visual hallucinations were reported, of which about half were well defined. All of them had no previous history of visual hallucinations and were under dopaminergic medication.

**Conclusions:**

The results show an inferior prevalence when comparing to the literature, albeit still frequent. Therapeutic adequacy has a well recognised impact at a functional level and prognosis. Therefore we stress the importance of a regular follow-up of these patients, recommending a rigorous and comprehensive clinical review.

